# Study on Utilization of Carboxyl Group Decorated Carbon Nanotubes and Carbonation Reaction for Improving Strengths and Microstructures of Cement Paste

**DOI:** 10.3390/nano6080153

**Published:** 2016-08-19

**Authors:** Xiantong Yan, Hongzhi Cui, Qinghua Qin, Waiching Tang, Xiangming Zhou

**Affiliations:** 1 Guangdong Provincial Key Laboratory of Durability for Marine Civil Engineering, College of Civil Engineering, Shenzhen University, Shenzhen 518060, China; yanxiantong@email.szu.edu.cn; 2 Research School of Engineering, Australian National University, Canberra 2601, ACT, Australia; qinghua.qin@anu.edu.au; 3 School of Architecture and Built Environment, the University of Newcastle, Callaghan 2308, NSW, Australia; patrick.tang@newcastle.edu.au; 4 Department of Mechanical, Aerospace and Civil Engineering, Brunel University London, Uxbridge, Middlesex UB8 3PH, UK; xiangming.zhou@brunel.ac.uk

**Keywords:** carbon nanotube, carbonation, functional group, cement hydration, calcium carbonate, crystal morphology, strength

## Abstract

Carbon nanotubes (CNTs) have excellent mechanical properties and can be used to reinforce cement-based materials. On the other hand, the reaction product of carbonation with hydroxides in hydrated cement paste can reduce the porosity of cement-based materials. In this study, a novel method to improve the strength of cement paste was developed through a synergy of carbon nanotubes decorated with carboxyl group and carbonation reactions. The experimental results showed that the carboxyl group (–COOH) of decorated carbon nanotubes and the surfactant can control the morphology of the calcium carbonate crystal of carbonation products in hydrated cement paste. The spindle-like calcium carbonate crystals showed great morphological differences from those observed in the conventional carbonation of cement paste. The spindle-like calcium carbonate crystals can serve as fiber-like reinforcements to reinforce the cement paste. By the synergy of the carbon nanotubes and carbonation reactions, the compressive and flexural strengths of cement paste were significantly improved and increased by 14% and 55%, respectively, when compared to those of plain cement paste.

## 1. Introduction

Though cement-based materials are the most extensively used construction material for various types of infrastructure all over the world, their major drawbacks such as low tensile strength, extreme quasi-brittleness, and the uncontrolled propagation of cracks have limited their engineering applications. To remove or alleviate the effect of these drawbacks, the concept of incorporating fine materials from the micro-scale to the nano-scale in cement paste has been realized in recent years [1,2]. As a desired reinforcing material, a carbon nanotube (CNT) is a tube-shaped material constructed with one-atom-thick rolled sheets of carbon and it belongs to an allotrope of carbon [2,3,4,5]. CNTs are usually classified as single-walled carbon nanotubes (SWCNTs) and multi-walled carbon nanotubes (MWCNTs) based on the concentric tube number [6]. Due to CNTs possessing excellent electrical and thermal conductivities [7,8], therefore, one of major reasons for using CNTs in cement-based materials is achieving multi-functionality. Carmen et al. [9] systematically studied the effect of CNT dosage, curing age, current intensity, loading rate and maximum stress applied on the strain-sensing properties of CNT-reinforced cement paste. They found that the cement-based CNT sensors showed more sensitive behavior at the age of 28 days, and the best performance as a strain sensor was obtained for the 0.05% CNT composites, reaching values of a gauge factor up to 240 with an *R*^2^ Pearson’s coefficient of 0.99. Other researchers [10,11,12,13,14] also did the similar studies.

To ensure the functions of CNT cement-based materials, suitable mechanical properties are necessary for applications. Table 1 summarizes the research from the literature about the positive effect of CNTs on the mechanical properties of cementitious composites. The improvement can be achieved by using different types of CNTs and adjusting the surface structure, aspect ratio, concentration and dispersion agent of CNTs.

It should be mentioned that the carbonation of cement-based materials can reduce the porosity of matrixes and increase the strength of the cementitious composites. Researchers have been using the carbonation curing method to improve the mechanical properties of cement-based materials [22,23]. 

Calcium carbonate (CaCO_3_) can be produced when cement hydration products are carbonated under a high CO_2_ concentration (higher than 10%) [24,25]. The chemical reactions of the carbonation of cement hydration products are listed as follows.

Ca(OH)_2_ + CO_2_ + H_2_O → **CaCO_3_** + 2H_2_O (1)

4CaO·Al_2_O_3_·13H_2_O + 4CO_2_ + H_2_O → 4**CaCO_3_** + 2Al(OH)_3_ + 11H_2_O (2)

3CaO·Al_2_O_3_·3CaSO_4_·31H_2_O + 3CO_2_ → 3**CaCO_3_** + 2Al(OH)_3_ + 3(CaSO_4_·2H_2_O) + 22H_2_O (3)

3CaO·2SiO_2_·3H_2_O + 3CO_2_ + nH_2_O → 3**CaCO_3_** + 2 SiO_2_aq + 3H_2_O (4)

It has been shown that the morphology of calcium carbonate crystals can be influenced by many factors such as positive ions, acids, surfactants, reactive conditions, etc. [26,27,28]. Some researchers utilized these parameters to achieve morphological control in calcium carbonate preparation or production [29,30,31,32]. The existing crystals can be in rhombic, spindle-like, or Nacre-like shapes. Spindle-like–shaped calcium carbonate crystals are preferred to be used in a reinforcing matrix. The shape of the calcium carbonate crystals can be controlled by a so-called “crystal control agent” and the pH value. A surfactant and the carboxyl group (–COOH) can serve as crystal control agents to influence the formation of calcium carbonate crystals. A concrete superplasticizer and CNTs or a CNT dispersion agent is a surfactant. Polycarboxylate superplasticizer (a kind of surfactant), a carboxyl group (–COOH, introduced into cement paste by CNTs), and different pH values of the samples influence the final morphology of the calcium carbonate crystals. 

## 2. Results and Discussion

Table 2 shows the compressive and flexural strengths of ordinary cement paste (CP) and cement paste with CNTs (CNT-CP) at 28 days and 56 days. For carbonated samples (C-CP and C-CNT-CP), the samples experienced 28 days standard curing followed by 28 days of carbonation before testing.

### 2.1. Effect of CNTs on Mechanical Properties and Microstructure of Cement Paste

From Table 2, it can be seen that the use of CNTs did not improve the compressive strength of cement paste effectively. The 28-day and 56-day compressive strengths of the CNT-CP samples were 39.4 MPa and 41.5 MPa, respectively, which was higher than those of the CP samples by 2%–3% only. However, the effect of CNTs on the flexural strengths of cement paste is very significant as the 28-day flexural strength increased by 34% (i.e., increased from 5.9 MPa to 7.9 MPa) compared to that of CP samples. The 56-day flexural strength of CP and CNT-CP were 6.2 MPa and 8.1 MPa, respectively. 

**Figure 1:**
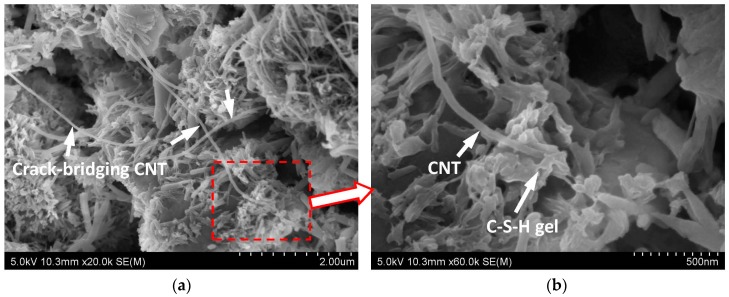
Scanning electron microscope (SEM) images of CNT-CP microstructures: (**a**) Microstructure of crack-bridging CNTs in CNT-CP unbroken sample; (**b**) A close-up image of the microstructure of the image in Figure 1a displaying the bond between the CNT and C-S-H gel (main product of cement hydration).

In order to investigate the underlying reason for this significant improvement in the flexural strength of CNT-CP, the microstructures of CNT-CP were studied by the scanning electron microscope (SEM). From the SEM images shown in Figure 1, the function of CNTs and their mechanisms in reinforcing the cement paste matrix can be revealed. Figure 1a shows the microstructure of the unbroken sample of CNT-CP. Although there were some cracks and pores in the cement paste matrix, the presence of CNTs could span the cracks or pores and connected the parts around the pores. Apparently the primary function of CNTs in cement paste is to reinforce the matrix through crack bridging which is very similar to what has been observed in fiber-reinforced matrixes. From Figure 1a, it can be seen that CNTs can bridge the micro-sized and nano-sized pores in cement paste. To achieve effective crack bridging by CNTs, a good bonding between the CNTs and cement paste matrix is essential and necessary. Figure 1b is a close-up of the microstructural photograph from a part of Figure 1a displaying the bond between CNTs and cement hydration products. From this figure, it can be clearly seen that the C-S-H gel, the main product of cement hydration, can wrap and fix the ends of CNTs firmly. As the diameter of CNTs is of nano-scale (~30 nm), CNTs can play an important role as nucleating agents for C-S-H gel, as reported in References [1,17,33]. Both the crack-bridging mechanism and good bonding between CNTs and the matrix can contribute to a remarkable improvement in the flexural strength of cement paste. Moreover, it is noted that CNTs can reduce the pores in the cement paste, which leads to an increase in the compressive strength of the cement paste.

**Figure 2:**
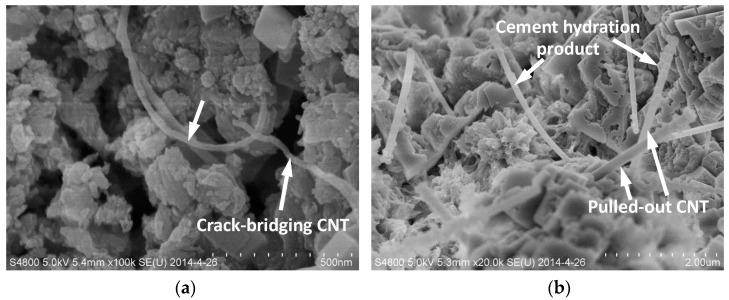
(**a**) Crack-bridging function of CNTs in a broken CNT-CP sample; (**b**) Pulled-out CNTs in a broken CNT-CP sample and the cement hydration product coated with CNT bundles.

Figure 2 shows the microstructures of the broken sample after the flexural test. The mechanisms of crack-bridging by CNTs and the pulled-out CNT can be seen in Figure 2a,b, respectively. The cement hydration product, i.e., C-S-H gel, coated along with the CNT bundles results in a denser microstructure and ultra-strong bonding between CNTs and the matrix. All the above-mentioned functions of CNTs have contributed to the significant improvement in the flexural strength of CNT-CP.

### 2.2. Synergy Effect of CNT and Carbonation on Mechanical Properties and Microstructure of Cement Paste

**Figure 3:**
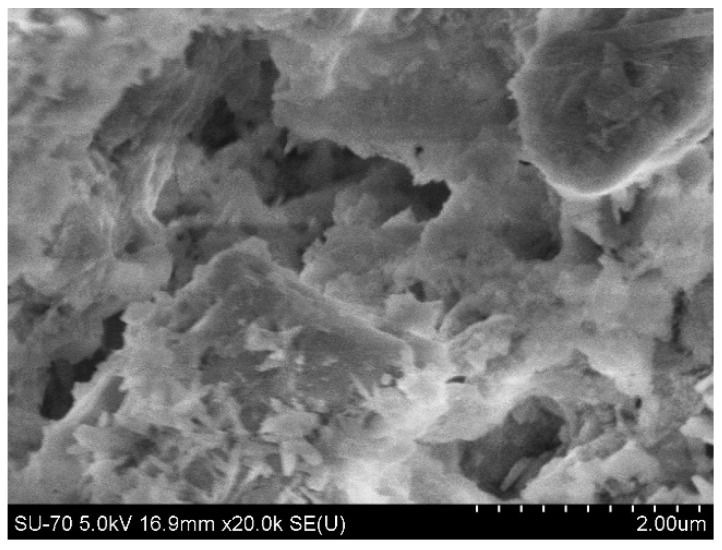
SEM image of the C-CP unbroken sample.

Figure 3 shows the SEM image of the microstructure of a C-CP unbroken sample. Compared with Figure 1, it is found that the pores in the cement paste have been filled by the carbonation products. Apparently the pores become less interconnected and more tortuous. This finding coincides with that of previous studies and confirms that carbonation can refine the pores of the cement paste matrix [3,6]. Due to the carbonation reactions, the porosity of the matrix is lower, resulting in the increase in the compressive strength of the cement-based materials. From Table 2, it can be seen that the compressive strength of the C-CP after carbonation curing was 44.9 MPa, which is higher than the 56-day compressive strength of CP (i.e., 40.1 MPa) by 12%. In the case of the flexural strength of the cement paste, a similar increase was observed for specimens that underwent carbonation curing, showing an increase of 10% in flexural strength. The results indicated that both the compressive and flexural strengths of cement paste can be improved due to carbonation and the effect of carbonation on the compressive strength is more significant than that on flexural strength. This is ascribed to the fact that the improvement in strength was mainly from the refinement of the pore structures and the compressive strength of the cement paste is more sensitive to the pore structure. 

**Figure 4:**
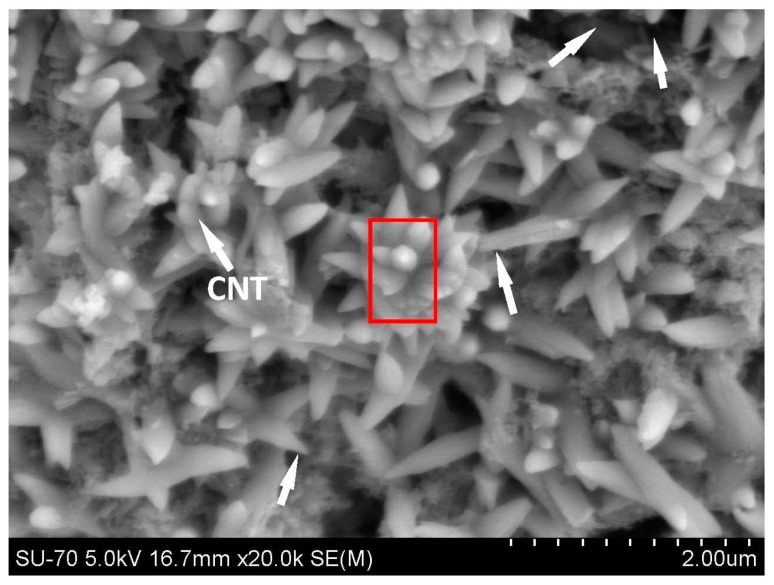
SEM image of C-CNT-CP unbroken sample.

Comparing the strength results of C-CNT-CP with those of CP, it can be clearly seen that the synergy of carbonation and CNTs can improve both the compressive and flexural strengths significantly. Particularly in case of flexural strength, the increase was as high as 55% and the strength increased from 6.2 MPa to 9.6 MPa. As seen in Table 2, the increase in flexural strength for CNT-CP specimens without carbonation curing was about 31% (i.e., from 6.2 MPa to 8.1 MPa). With carbonation curing, the flexural strength of C-CNT-CP was further enhanced (i.e., from 8.1 MPa to 9.6 MPa) with an additional increase of 19% compared to CNT-CP specimens. In this work, the surfactant (polycarboxylate superplasticizer) and carboxyl group (–COOH) served as crystal control agents to influence the formation of calcium carbonate crystals in carbonated cement-based materials. The significant improvement in flexural strength for C-CNT-CP specimens is mainly ascribed to their microstructural change. Figure 4 shows the SEM image of the C-CNT-CP unbroken sample after carbonation curing. It can be clearly shown that the microstructure of the C-CNT-CP is totally different from that of the CNT-CP as its microstructure was characterized by lots of spindle-like crystals in the cement paste. Some CNTs can be found among the spindle-like crystals (see Figure 4). In order to confirm that the spindle-like crystals are calcium carbonate crystals, an energy-dispersive X-ray spectrometer (EDS) was employed, which was utilized to scan the area of the red box as indicated in Figure 4 for investigating the main elements of the spindle-like crystals. Based on the main elements, the category of the spindle-like crystal can be deduced. The results of the EDS analysis are listed in Table 3. From the results, it can be known that C (carbon), O (oxygen) and Ca (calcium) are the three main elements of the spindle-like crystals. Therefore, the main composite of the spindle-like crystals should be calcium carbonate (CaCO_3_). The lower-content elements of Al (alumina), Si (silica) and S (sulphur) indicated the materials of carbonated/uncarbonated cement hydration products (see Equations (2)–(4)).

**Figure 5:**
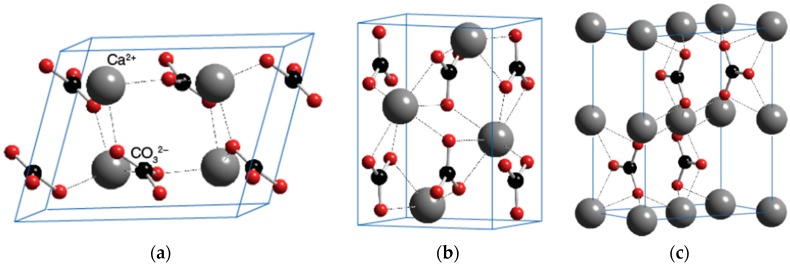
Crystal structures of the polymorphs of calcium carbonate. (**a**) Calcite; (**b**) Aragonite; (**c**) Vaterite.

CaCO_3_ has three different polymorphs: calcite, aragonite and vaterite. Calcium carbonate’s most common and stable form is the hexagonal calcite. The orthorhombic aragonite is less abundant, and the hexagonal vaterite is the least common of the three polymorphs [34]. Figure 5 shows the crystal structures of the polymorphs of calcium carbonate. According to the previous studies [35,36], the spindle-like crystal of CaCO_3_ mainly consists of calcite polymorphs. 

**Figure 6:**
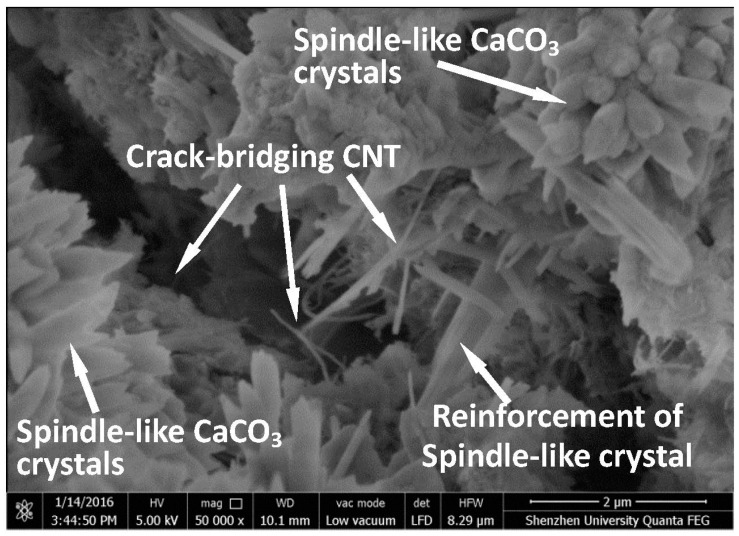
SEM image of C-CNT-CP broken sample.

Figure 6 presents the microstructure of the broken C-CNT-CP sample after the flexural test. From this figure, the synergy effect of CNTs and spindle-like CaCO_3_ crystals on the flexural strength of cement paste can be clearly seen. The CNTs, as identified earlier, played the role of crack bridging in cement paste. On the other hand, more importantly, the spindle-like CaCO_3_ crystals (carbonated crystals) were found to cross the micro-cracks and reinforce the microstructure of the cement paste. The function of spindle-like CaCO_3_ crystals is similar to a reinforcing steel bar in concrete. Therefore, the flexural strength of C-CNT-CP was improved dramatically.

## 3. Materials and Methods

### 3.1. Materials

**Figure 7:**
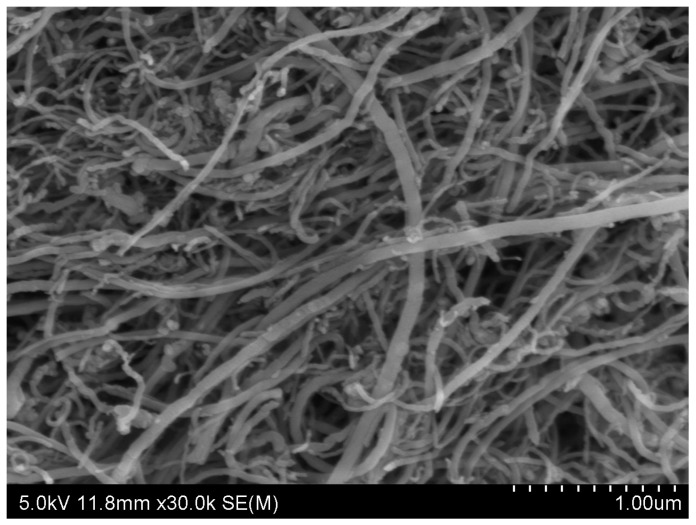
Undispersed carbon nanotubes (CNTs).

Industrial grade hydroxyl multi-wall carbon nanotubes (MWCNTs) used in this study (see Figure 7) were supplied by the Physics Institute of Chinese Academy of Sciences (Mianyang, Sicuan, China). The properties of the MWCNTs are enlisted in Table 4. 

The cement used in the experiments was ordinary portland cement (OPC) that conformed to BS EN 197-1:2001, with a 28-day mortar compressive strength of 57 MPa. The specific gravity of the cement was 3.4 g/cm^3^ and its fineness was 3950 cm^2^/g. The initial and final setting times were 155 min and 8 h 15 min, respectively. The oxide compositions are given in Table 5.

The superplasticizer used in this research is a polycarboxylate-based superplasticizer (PC). PC is a kind of surfactants and it contains both active polar groups and non-polar groups. Except polar groups which can attach on the cement particles in fresh cement paste, non-polar groups can be absorbed on the surface of the CNTs by the –COOH group. Therefore, the PC was used to disperse the CNTs in cement matrix.

### 3.2. Sample Preparations

CNT suspension was prepared by mixing 1 g CNTs with 0.2 g PC in 99 g aqueous solution under ultrasonication for 30 min using a 3 mm tip diameter probe-type sonicator with 20 kHz frequency and 650 W ultimate power. The input power of the sonicator was fixed at 165 W (i.e., 30% of its ultimate capacity). To reduce the temperature increase, the suspensions were cooled in a water–ice bath for 5 min in every 10 min of ultrasonication.

The details of the mix design are shown in Table 6. The superplasticizer is polycarboxylate-based water reducing admixture. As the CNTs were contained into the CNT suspension, the total CNT suspension was calculated based on the CNTs quantity.

CNT suspension and polycarboxylate superplasticizer were mixed into cement paste with a water-to-cement ratio of 0.35. The CNT dosage was 0.25% by mass of cement. In the process of mixing CNT-CP, the water, superplasticizer and CNT suspension were added into cement and mixed for 3 min at low-speed and then another 1 min at high-speed. After the mixing was complete, the mixture was poured into moulds and compacted on a vibration table. All the specimens were covered with a plastic film to prevent water loss and demolded after 24 h. After demolding, the specimens were cured in a curing room (20 ± 1 °C and 98% RH (Relative Humidity)) for 28 days. A total of 24 samples (three samples per group) with dimensions of 40 × 40 × 160 mm were prepared for determining the mechanical properties (flexural and compressive strengths) at corresponding test ages (28 days and 56 days). The corresponding results are shown in Table 2. 

### 3.3. Carbonation Curing Method

Due to the fact that cement hydration products can react with CO_2_ within a suitable humidity range, it is believed that the highest rate of carbonation is in the range of RH about 60%–70% as reported by [13]. In this research, the accelerated carbonation method according to GB/T50082-2009 was adapted and the 40 mm × 40 mm × 160 mm specimens were placed in a carbonation chamber for 28 days before the mechanical tests. In the chamber, the CO_2_ concentration was 20% and the RH was 70%. According to previous research [12], cementitious sample with water/cement ratio of 0.4 could be fully carbonated after 28 days accelerated carbonation curing.

### 3.4. Test Methods

#### 3.4.1. Strength

In this research, the mechanical properties (compressive and flexural strength) of carbonated (i.e., C-CP and C-CNT-CP) and uncarbonated (i.e., CP and CNT-CP) samples were measured at the age of 28 and 56 days. Test prisms with the dimensions of 40 mm × 40 mm × 160 mm were used to study the mechanical properties of CNT-CP. The loading rate of 50 ± 10 N/s and 2400 ± 200 N/s for the flexural and compressive strength tests was used, respectively. The load tests were performed in compliance with the ISO 679:1989 and GB/T 17671-1999 standards (Methods of testing cements—Determination of strength). 

#### 3.4.2. Microstructure

Microstructure of cement paste was examined using Hitachi Su-70 field emission-scanning electron microscope (FE-SEM, Tokyo, Japan) at an accelerating voltage of 1.0 kV. SEM sample was picked from the cement paste and then dried in the oven at 100 °C for 24 h. SEM analysis was carried out once the oven-dried sample was cooled down to room temperature. The samples were then sprayed with gold prior to observation. In order to investigate elements of the products in the prepared samples, the energy-dispersive X-ray spectrometer (EDS, Tokyo, Japan) was employed.

## 4. Conclusions

(1) The effect of CNTs on the flexural strength of cement paste is more significant than that on the compressive strength. The 28-day compressive and flexural strengths of CNT-CP specimens increased by 2% and 34%, respectively, when compared to those of CP samples without CNTs.
(2) The SEM results showed that the function of CNTs in cement paste is to reinforce the matrix through crack bridging and the CNTs can also bridge the micro-sized and nano-sized pores. Both the crack-bridging mechanism and good bonding between the CNTs and matrix can contribute to a remarkable improvement in the flexural strength of cement paste.
(3) The polycarboxylate superplasticizer and carboxyl group (–COOH) can serve as crystal control agents to influence the spindle-like formation of calcium carbonate crystals in carbonated cement-based materials.
(4) The synergy of carbonation and CNTs can improve both the compressive and flexural strengths of cement paste significantly. In the case of the flexural strength, the increase was as high as 55%.
(5) EDS analysis confirmed that the spindle-like crystals are calcium carbonate (CaCO_3_). The SEM results showed that the spindle-like CaCO_3_ crystals can cross the micro-cracks and reinforce the microstructure of cement paste.

## Figures and Tables

**Table 1:** Improvement of mechanical properties of cementitious composites with carbon nanotubes (CNTs). SWCNTs: Single-walled carbon nanotubes.

Properties	Improvement	CNTs			Surfactant	Researchers
		Length	Diameter	Concentration		
Compressive strength	0	10 μm	10 nm	0.007–0.042 wt %	polyacrylic acid polymers	Cwirzen [15]
	11%	-	8 nm	0.02%	Nil	Morsy [16]
	19%	30 μm	2 nm	0.1% f-SWCNT	Pluronic F-127	Parveen [17]
Flexural strength	0	10 μm	10 nm	0.007–0.042 wt %	polyacrylic acid polymers	Cwirzen [15]
	47%	-	-	0.25 wt %	Polyvinylpyrrolidone	Chan [18]
	25%	30 μm	40 nm	0.08 wt %	surfactant	Konsta-Gdoutos [13]
	25%	100 μm	40 nm	0.048 wt %	surfactant	Konsta-Gdoutos [13]
	36%	30 μm	20 nm	0.26 wt %	surfactant	Metaxa [19]
	269%	1.5 μm	9.5 nm	0.2 wt %	polycarboxylate	Luo [20]
	65%	30 μm	8 nm	0.1 wt %	polycarboxylate	Luo [20]
	50%	1.5 μm	9.5 nm	0.075 wt %	polycarboxylate	Zou [21]

**Table 2:** Compressive and flexural strengths of carbonated and uncarbonated samples with or without CNT.

Items	Compressive Strength (MPa)						Flexural Strength (MPa)					
	28 Days			56 Days			28 Days			56 Days		
	Tested Date	Average	Standard Deviation	Tested Date	Average	Standard Deviation	Tested Date	Average	Standard Deviation	Tested Date	Average	Standard Deviation
**CP**	39.1	38.7	0.76	40.1	40.1	0.78	6.3	5.9	0.29	6.6	6.2	0.29
	39.3			39.2			5.6			6.1		
	37.6			41.1			5.8			5.9		
**CNT-CP**	40.3	39.4	0.67	39.9	41.5	1.14	8.4	7.9	0.37	7.9	8.1	0.33
	38.7			41.9			7.9			7.9		
	39.2			42.6			7.5			8.6		
*Ratio of CNT-CP/CP*	*/*	*1.02*	*/*	*/*	*1.03*	*/*	*/*	*1.34*	*/*	*/*	*1.31*	*/*
	**28 Days**			**28 Days Curing + 28 Days Carbonation**			**28 Days**			**28 Days Curing + 28 Days Carbonation**		
	**Tested Date**	**Average**	**Standard Deviation**	**Tested Date**	**Average**	**Standard Deviation**	**Tested Date**	**Average**	**Standard Deviation**	**Tested Date**	**Average**	**Standard Deviation**
**C-CP**	/	/	/	45.4	44.9	1.87	/	/	/	6.7	7.0	0.34
	/			46.9			/		/	6.9		
	/			42.4			/		/	7.5		
**C-CNT-CP**	/	/	/	46.2	45.7	0.56	/	/	/	10.2	9.6	0.49
	/			45.9			/		/	9.0		
	/			44.9			/		/	9.6		
*Ratio of C-CP/CP*	*/*	*/*	*/*	*/*	*1.12*	*/*	*/*	*/*	*/*	*/*	*1.13*	*/*
*Ratio of C-CNT-CP/CP*	*/*	*/*	*/*	*/*	*1.14*	*/*	*/*	*/*	*/*	*/*	*1.55*	*/*
*Ratio of C-CNT-CP/CNT-CP*	*/*	*/*	*/*	*/*	*1.16*	*/*	*/*	*/*	*/*	*/*	*1.19*	*/*

**Table 3:** Energy-dispersive X-ray spectrometer (EDS) results of C-CNT-CP sample. MDL: Method Detection Limit.

Element	Line	Atomic (%)	Atomic Ratio	Concentration (wt %)	Error 2-Sigma	MDL 3-Sigma
**C**	**Ka**	**10.22**	0.18	5.53	0.286	0.276
**O**	**Ka**	**57.40**	1.00	41.35	0.802	0.378
Al	Ka	1.49	0.03	1.81	0.080	0.086
Si	Ka	8.25	0.14	10.44	0.138	0.079
S	Ka	0.77	0.01	1.12	0.065	0.075
K	Ka	0.21	0.00	0.38	0.054	0.073
**Ca**	**Ka**	**21.22**	0.37	38.29	0.274	0.084
Fe	Ka	0.44	0.01	1.10	0.110	0.131
Total		100.00		100.00		

**Table 4:** Properties of the multi-wall carbon nanotubes (MWCNTs).

Items	Data
Outer Diameter	30–50 nm
–COOH Content	0.6%
Length	20–30 μm
Purity	>90 wt %
Ash	<8 wt %
Specific Surface Area (SSA)	40 m^2^/g

**Table 5:** Compositional analysis expressed as oxides (wt %) of cement.

Oxides (wt %)	CaO	SiO_2_	Al_2_O_3_	Fe_2_O_3_	K_2_O	MgO	TiO_2_	Si/Ca
Ordinary portland cement (OPC)	64.6	21.10	5.90	3.10	-	1.00	-	0.327

**Table 6:** Details of mix proportion.

Type of Cement Paste	Cement	Water	CNTs	Superplasticizer
Cement Paste (CP)	1	0.40	0%	0.30%
CNT-CP	1	0.40	0.25%	0.40%

## Abbreviations

CNT	Carbon nanotube
CP	Cement paste
C-CP	Carbonated CP
CNT-CP	CNT reinforced CP
C-CNT-CP	Carbonated CNT-CP
