# Correction: Yan, X., et al. Study on Utilization of Carboxyl Group Decorated Carbon Nanotubes and Carbonation Reaction for Improving Strengths and Microstructures of Cement Paste. *Nanomaterials* 2016, *6*, 153

**DOI:** 10.3390/nano6100185

**Published:** 2016-10-13

**Authors:** Xiantong Yan, Hongzhi Cui, Qinghua Qin, Waiching Tang, Xiangming Zhou

**Affiliations:** 1 Guangdong Provincial Key Laboratory of Durability for Marine Civil Engineering, College of Civil Engineering, Shenzhen University, Shenzhen 518060, China; yanxiantong@email.szu.edu.cn; 2 Research School of Engineering, Australian National University, Canberra 2601, ACT, Australia; qinghua.qin@anu.edu.au; 3 School of Architecture and Built Environment, the University of Newcastle, Callaghan 2308, NSW, Australia; patrick.tang@newcastle.edu.au; 4 Department of Mechanical, Aerospace and Civil Engineering, Brunel University London, Uxbridge, Middlesex UB8 3PH, UK; xiangming.zhou@brunel.ac.uk

The authors wish to make the following correction to this paper [1].

The published Figure 3 was incorrect. The correct Figure 3 is shown below.

The authors regret any inconvenience or misunderstanding caused by this error. The manuscript will be updated and the original will remain available on the article webpage.

## Figures and Tables

**Figure 3:**
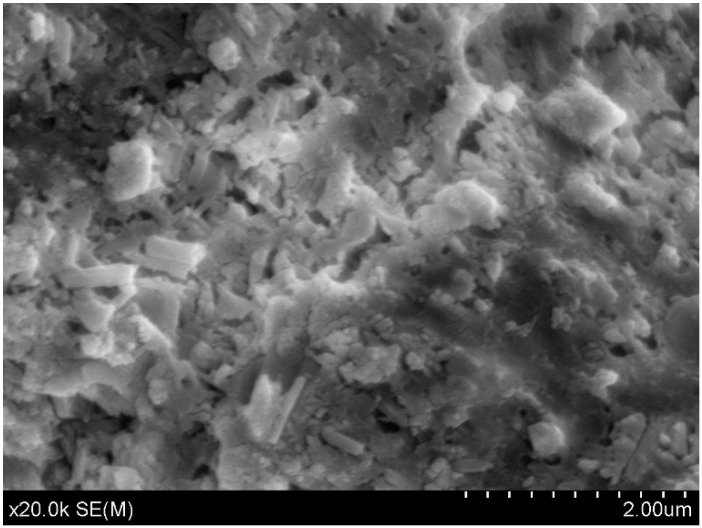
SEM image of the C-CP unbroken sample.
